# A New Cold-Active Glucose Oxidase From Penicillium: High-Level Expression and Application in Fish Preservation

**DOI:** 10.3389/fmicb.2020.606007

**Published:** 2020-11-23

**Authors:** Mingxue Yuan, Chen Ning, Suxiao Yang, Qingping Liang, Haijin Mou, Zhemin Liu

**Affiliations:** College of Food Science and Engineering, Ocean University of China, Qingdao, China

**Keywords:** glucose oxidase, cold-active, *Pichia pastoris*, high-efficiency expression, antimicrobial, fish preservation

## Abstract

Glucose oxidase (GOx) with high enzyme activity at low temperature (4°C) is potentially useful for food preservation, especially for aquatic products preservation. A cold-active GOx with approximately 83% similarity to known protein sequences, was isolated from *Penicillium* sp. MX3343 and expressed in *Pichia pastoris* X33. Through high cell density fermentation, the yield of recombinant enzyme (named GOxP_5_) reached 458.6 U/mL. GOxP_5_ showed optimal activity at 30°C and pH 5.5, and was stable at a broad pH range from pH 2–6. Moreover, GOxP_5_ could maintain 72% maximum activity at 4°C, suggesting its application for the preservation of aquatic products at low-temperatures. Importantly, GOxP_5_ showed a good antimicrobial effect against common fish pathogenic bacteria (*Listeria monocytogenes* and *Vibrio parahaemolyticus*). Moreover, sensory, microbiological (total bacterial count), and physicochemical (total volatile basic nitrogen and pH) systematic analyses proved GOxP_5_ to be an excellent freshness preserving agent in the context of the grass carp. These favorable enzymatic properties of GOxP_5_ make it potentially useful in food biopreservation, and the effect was better compared to the commonly used chemical preservatives.

## Introduction

Glucose oxidase (GOx; glucose aerodehydrogenase; E.C.1.1.3.4.) is a flavoprotein that catalyzes the dehydrogenation of β-D-glucose to gluconic acid and hydrogen peroxide (H_2_O_2_) using molecular oxygen as an electron acceptor and FAD acts as a redox carrier (Hatzinikolaou et al., 1996; Bankar et al., 2009). GOx attracted much attention due to its wide applications in the biosensors, biofuel, chemical, beverage and animal feed industries, as well as in the food preservation field (Field et al., 1986; Gao et al., 2012; Tang et al., 2016). Such applications include its use in bread to strengthen gluten, in glucose sensors to monitor the environment, in feed to improve the intestinal acid digestive environment, and even in food preservation to extend shelf life.

In the food preservation industry, biopreservatives such as chitosan (Jeon et al., 2002), antibacterial peptides (e.g., nisin), lysozyme (Sun et al., 2020), and glucose oxidase (Dondero et al., 1993), have a safer and better effect on delaying the spoilage time of food compared to traditional chemical preservatives. GOx was used as a preserving agent with a significant antibacterial effect against *Pseudomonas fragi* (Yoo and Rand, 1995), enterotoxigenic *Escherichia coli*, *L. monocytogenes*, and *V. parahemolyticus* in aquatic products (Massa et al., 2001); of note, these bacteria are associated with food contamination. However, the applications of GOx in the context of the preservation of aquatic products are limited owing to the absence of cold-active enzymes. Importantly, GOx are widely found in nature and have been purified from different microbial sources for commercial applications; these sources include fungi such as *Penicillium* and *Aspergillus*, especially *P. amagasakiense* and *A. niger* (Guo et al., 2010; Gao et al., 2012). Of note, GOx from *Penicillium* showed better catalytic efficiency for glucose oxidation (Kusai et al., 1960). However, few of the reported GOx could retain the required enzymatic activity at 4°C.

An ideal GOx for the preservation of aquatic products should possess three properties: cold-active ability, acid and alkali resistance, and highly specific activity. Studies have reported the use of GOx to extend shelf life of shrimp products. Dondero et al. (1993) found that shrimps maintained in a GOx/Catalase/glucose solution could be stored for up to 13 days before being refrigerated compared to 6 days for control shrimp preparations. Moreover, Xu et al. (2018) improved the quality and freshness of shrimps subjected to cold storage via the treatment with a novel GOx (12.5 U/mL) from *Bacillus* sp. CAMT22370. However, little information about the use of GOx to preserve fish is available. As a freshwater fish, grass carp is easily susceptible to spoilage; therefore, it is particularly important to maintain its freshness via the use of preservatives such as GOx.

Here, a new gene encoding a cold-active GOx from *Penicillium* sp. MX3343 was acquired, and expressed heterologously in *Pichia pastoris* (Macauley-Patrick et al., 2005). The biochemical characteristics of the recombinant enzyme were analyzed, and the yield of GOx was significantly enlarged via high cell fermentation. Moreover, for better evaluating its potential in preserving aquatic products, two common fish pathogens were selected to test its antibacterial effect. Subsequently, a fresh-keeping experiment using grass carp at 4°C was performed to prove its potential as a low-temperature food preservative agent.

## Materials and Methods

### Materials

The GOx gene was cloned from *Penicillium* sp. MX3343, which we isolated and identified from soil. The pPICZα A was used as an expression vector to construct recombinant plasmids with coding genes (pPICZα A-GOxP_5_, pPICZα A-proGOxP_5_). *Escherichia coli* DH5α (Sangong, China) and *Pichia pastoris* X33 (Invitrogen, United States) were used as the expression and propagation hosts, respectively. All other chemicals in this research were of analytical grade.

### Cloning and Sequencing of the GOx Gene

Genomic DNA was extracted using the CTAB extraction method described by Van Burik et al. (1998) from *Penicillium* sp. MX3343 culture. The three specific primers, GOxF1, GOxF2, and GOxR (Table 1) were designed and synthesized based on *Penicillium* GOx transcriptome analysis. Genomic DNA was used as a template, and GOxF1, GOxF2, and GOxR with an annealing temperature of 56°C were used as primers for PCR amplification to amplify the full-length sequences of GOxP_5_ and proGOxP_5_. The PCR products were purified, linked to the pPICZα A and sent to Beijing Ruiboxingke Co., Ltd. for sequencing. Signal peptides were analyzed using Signal 4.0 Server. A BLAST server^1^ was used for DNA and protein sequence alignment and conserved domain analysis. The GOxP_5_ nucleotide sequence used in this study was submitted to GenBank (Accession No. MN912809).

**TABLE 1:** Primers used in this study.

Primers	Sequence 5′–3′
GOxF1	CAGGGCTTCACTCCAGCCG
GOxF2	ATGAAGTCCATCATTCTTGCCTC
GOxR	TTAATGATGATGATGATGATGGGGCTTGTAGTCAGCCAGAACA

### High-Level Expression of the GOx Gene and High Cell Density Fermentation

The recombinant plasmids pPICZα A-GOxP_5_ and pPICZα A-proGOxP_5_ were linearized using *Sac*I and transformed into *P. pastoris* X33 using electroporation with a Gene Pulser device (Invitrogen, United States) based on the manufacturer’s instructions. Zeocin was used as selection marker, and the liquid-transformed vector amplification (PTVA) method was used to screen multi-copy strains with high enzyme activity (Aw and Polizzi, 2016). The resultant transformants were inoculated into YPD medium containing zeocin and cultivated at 30°C. With treatment every 12 h and transfer to the next medium containing a higher concentration of antibiotics. After colonies were observed, they were numbered in order and inoculated into a 48-well-deep plate containing BMGY liquid medium for fermentation. To maintain induction, 100% (v/v) methanol was added to the growing culture during selection to a final concentration of 1% (v/v) every 24 h. The strain with the highest enzyme activity was selected and used in subsequent experiments.

The GOxP_5_ strain was selected to high cell density fermentation in a 20 L fermentor. First of all, the strain was cultured based on previous research (Liu et al., 2020a). Secondly, the shake flask culture solution was transferred to the fermenter containing 10.0 L of basic salt medium and 40 mL PTM 1 trace salt. The temperature and pH of the fermentation process were maintained at 30°C and 6.0, and the rotation speed was controlled. Finally, fed-batch process was carried out when the wet cell mass reached 180 g/L, and the rate was adjusted during the methanol induction stage to keep the dissolved oxygen concentration above 25%. The unit of methanol addition rate is mL/L/h, which referred to the amount of methanol that needs to be added per hour in a 20 L fermentor. The wet cell mass, enzyme activity and protein content of the sample were measured at different time points throughout the induction phase.

### Purification of GOxP_5_ and proGOxP_5_

The fermentation mixture was centrifuged at 10,000 × g for 10 min to remove yeast pellet. The fermented supernatant was concentrated and ultrafiltrated by using a 50 kDa cut-off membrane. According to the previous study (Uchima et al., 2012), a Ni-sepharose 6FF column (GE Healthcare, United States) was used to purify the crude enzyme solution sample. SDS-PAGE (12.5% running gel) was performed to analyze the GOxP_5_ molecular weight distribution. According to the Bradford (1976), the protein concentration was determined at 595 nm with bovine serum albumin (BSA) as the standard.

### Biochemical Characterization of GOxP_5_ and proGOxP_5_

The enzyme activity of GOxP_5_ was determined based on the method in previous research (Guo et al., 2010). After 3 mL reaction system was incubated in a 30°C water bath for 5 min, 0.1 mL enzyme solution was added, and the absorbance change value in 1 min was measured at 500 nm. The amount of enzyme required to oxidize 1 μmol of D-glucose into D-gluconic acid and H_2_O_2_ per minute is defined as one unit of GOxP_5_ activity. The optimum temperature for GOxP_5_ and proGOxP_5_ activity was assayed by assessing the relative activities at different temperatures (4–70°C) in sodium acetate buffer (50 mM, pH 6.0). To estimate thermostability, the residual activity of GOxP_5_ was assayed after preincubation in sodium acetate buffer (50 mM, pH 6.0) at several temperatures (5–60°C) for 10 min, followed by cooling on melting ice for 10 min.

The optimum pH for GOxP_5_ and proGOxP_5_ activity was assayed by three different buffers with used as follows: pH 2.0–3.0 (0.05 M glycine-HCl), pH 4.0–6.0 (0.05 M NaAc-HAc), and pH 7.0–8.0 (0.05 M Tris-HCl). To investigate pH stability, GOxP_5_ was detected after preincubation in different pH buffers (pH 2.0–8.0) at 25°C for 2 h before assaying for residual activity.

Thirteen metal ions and chemical reagents were selected to investigate their effects on the activity of recombinant GOxP_5_ and proGOxP_5_ based on the method described by our previous study (Liu et al., 2020b). The group without the additional metal ions and chemical reagents was used as the control. In addition, the substrate specificity was analyzed under the conditions of 30°C and pH 6.0. The effect of different substrates on the activity of purified GOxP_5_ and proGOxP_5_ was evaluated by measuring the activity in the presence of 18 mg/mL of various substrates. The group with glucose was used as the control.

The kinetic parameters of GOxP_5_ and proGOxP_5_ were assayed in 0.1 M sodium phosphate (pH 6.0) with β-D-glucose (50–2,000 mM) as substrate at 30°C. According to the glucose oxidase determination standard QWSY 01-2010 in China and the study by Guo et al. (2010), the reaction was measured within 2 min with sufficient oxygen. The data were determined by fitting the Michaelis–Menten plot using non-linear regression with the software Origin 8.0.

### Docking Analysis of GOxP_5_ and β-D-Glucose

Automatic homology modeling of GOxP_5_ was performed using the Phyre2 program^2^ (Kelley et al., 2015). The tertiary structure of GOxP_5_ was docked with its substrate β-D-glucose using AutoDock Tools 1.5.6. The most reasonable docking complex was chosen according to the guidelines mentioned in previous reports (Trott and Olson, 2010). PyMOL software (v1.7.2; Delano Scientific, United States) was used to visualize three-dimensional structures and prepare figures.

### Antibacterial Evaluation of GOxP_5_

The Oxford cup method (Wang et al., 2011) was used to determine the antibacterial effect of recombinant GOxP_5_ on five bacteria including *Escherichia coli* (ATCC 25922), *Salmonella derby* (ATCC 13076), *Staphylococcus aureus* (ATCC 6538), *Vibrio parahaemolyticus* (ATCC 17802), and *Listeria monocytogenes* (ATCC 19115). Each bacterium was cultivated and adjusted to 10^7^ CFU/mL, spread on petri dishes of LB or TSB medium. Based on the study of Liu et al. (2020c), the Oxford cups were put on the culture medium, 150 μL recombinant GOxP_5_ solution (with 2% glucose added) with enzyme activity of 1 and 2 U/mL, respectively, were added to the Oxford cups. Sterile normal saline solution was used as a control. Finally, the strains were incubated at 37 ± 1°C for 24 h.

Furthermore, the antibacterial activity of GOxP_5_ (with 2% glucose added) was evaluated by measuring the minimum inhibitory concentration (MIC) and the minimum bactericidal concentration (MBC) by the broth dilution method (Başyiǧit et al., 2020). The effect of the GOxP_5_ on growth curves of *L. monocytogenes* and *V. parahemolyticus* were studied by adding GOxP_5_ with different concentrations of 4 × MIC, 2 × MIC, 1 × MIC, 1/2 × MIC, 1/4 × MIC, and sterile normal saline solution was used as control.

### SEM Observation

The *L. monocytogenes and V. parahemolyticus* were cultured to 10^7^ CFU/mL, incubated with MIC concentration of GOxP_5_ (with 2% glucose added) for 2 h. The samples suspended in PBS were then fixed in 2.5% glutaraldehyde solution and placed at 4°C. The fixed samples were observed by Scanning Electron Microscopy (SEM) with 20 kV of acceleration voltage after corresponding processing (Asad et al., 2020).

## Fish Preservation Experiment

### Fishes Collection and Treatment

Five live grass carps weighing approximately 1 kg each were purchased from the Tuandao aquatic market in Qingdao, China, and immediately transported alive in water to the laboratory within 3 h. In the laboratory, we first percussive stunning, then fish were killed by sectioning the spinal cord at the base of the head immediately, to meets the requirements of ethical standards (López-Luna et al., 2013). All fish experiments has been carried out in accordance with European Food Safety Authority (2009) and EU Directive 2010/63/EU. The grass carps were stunned, scaled, gutted, decapitated, deboned, and peeled manually before immediately cleaning and washing with saline water. Each fish was divided into 10 pieces, each piece weighing between 30 and 40 g. The GOxP_5_ (12.5 U/mL) was diluted with sterile water to 1 U/mL according to Field et al. (1986) and Dondero et al. (1993). Then, the samples were randomly assigned to four treatments: the filets were drenched in 1.25% SBS (sodium bisulfite), 0.5% Vc (Vitamin C, Ascorbic Acid), or 1 U/mL GOxP_5_ for 10 min. Control samples were immersed in sterile water, removed and drained for 30 min after dipping. Each filet was packaged in polyethylene (PE) packaging pouches, sealed, and stored at 4°C. All operations were performed on a UV-cleaned bench. Experiments were performed in triplicate for the sensory analysis, microbiological analysis [total bacterial count (TBC)], and physicochemical analysis [total volatile basic nitrogen (TVB-N) and pH] of the grass carp filets; these experiments were conducted every second day at 4°C for samples stored over a 10 days period.

### Sensory Analyses

Sensory analyses were performed using the method recommended by Ojagh et al. (2010) with some modifications; these analyses were performed by six rigorously trained laboratory panelists, and culculate of overall sensory score based on the Supplementary Table 2. Four parameters (texture, odor, color, and elasticity) were used to evaluate fish filet quality on a score from 1 to 10, with 10 performing the best in terms of freshness and the score decreasing as the quality of the filets gradually decreased. Total sensory score result of 9 represents absolutely fresh, 7 represents acceptable, and 5 represents unacceptable.

### Microbiological Analysis

The total bacterial count (TBC) was determined from plate count agar. Each weighed fish filet sample (5 g) was added to 45 mL sterile physiological saline water (0.85%) and homogenized for 1 min; this was serially diluted with the same volume of normal saline water. Appropriate dilutions (1 mL) were poured into a petri dish and mixed with plate count agar medium (incubated at 30 ± 1°C for 72 h).

### Physicochemical Analysis

Determination of total volatile basic nitrogen (TVB-N, mg/100 g sample) values was based on the current Chinese food safety standard method (GB 5009.228-2016) via a micro-diffusion method. A 10 g minced fish sample was dispersed in 50 mL of distilled water and filtered after 30 min of immersion. Then, 1 mL filtrate was mixed with 1 mL saturated K_2_CO_3_ in a diffusion dish. Additionally, 1 mL distilled water was used instead of the filtrate as a control. After 2 h in an incubator at 37 ± 1°C, the distillate was collected into the center of a diffusion dish containing 1 mL of 25 g/L boric acid and a mixed indicator. The distillate was titrated to calculate the result. A 10 g fish sample was ground using a JR-22 experimental meat grinder (Zhucheng HSBC Food Machinery Co., Ltd., Shandong, China) and determined by a digital pH meter (Horiba, LAQUAtwin-pH-22, Japan).

### Data Analysis

All analyses were carried out in triplicate. The data were statistically analyzed using Origin 8.0 procedures, and the least significant difference (LSD) procedure was used to test significance using a significance threshold of *p* < 0.05.

## Results

### Gene Cloning and Sequence Analysis

The proGOxP_5_ precursor gene consists of 1,818 bp, and the first 16 amino acids were identified using Signal 4.0 server prediction as a signal peptide. No introns were identified in the sequence. The mature protein is 590 amino acids long, and it has a calculated molecular weight (Mw) of 63.86 kDa; the Isoelectric point (pI) was pH 5.05. GOxP_5_ was most similar to *Penicillium subrubescens* CBS 132785 (GenBank accession No.: OKO89536.1) based on the amino acid sequence in GenBank, exhibiting 83.78% identity. The two active sites His540 and His583 perform the roles of proton donor and proton acceptor, respectively (Supplementary Figure 1).

### High-Level Expression in *P. pastoris* X33 and High-Cell-Density Fermentation

The optimized GOxP_5_ and proGOxP_5_ genes (with the signal peptide coding sequences) were introduced into the expression vector pPICZα A and independently transformed into *P. pastoris* X33-competent cells. The *P. pastoris* recombinant strain (X33/GOx) with high enzyme activity was picked out via a three-round selection. The positive transformants showing the highest enzyme activity were screened from a total of 1,000 positive clones. After induction with methanol for 72 h, the highest GOxP_5_ and proGOxP_5_ activities reached 35 and 7 U/mL, respectively. Cultures of the control strain X33/pPICZα A did not exhibit GOx activity.

**FIGURE 1:**
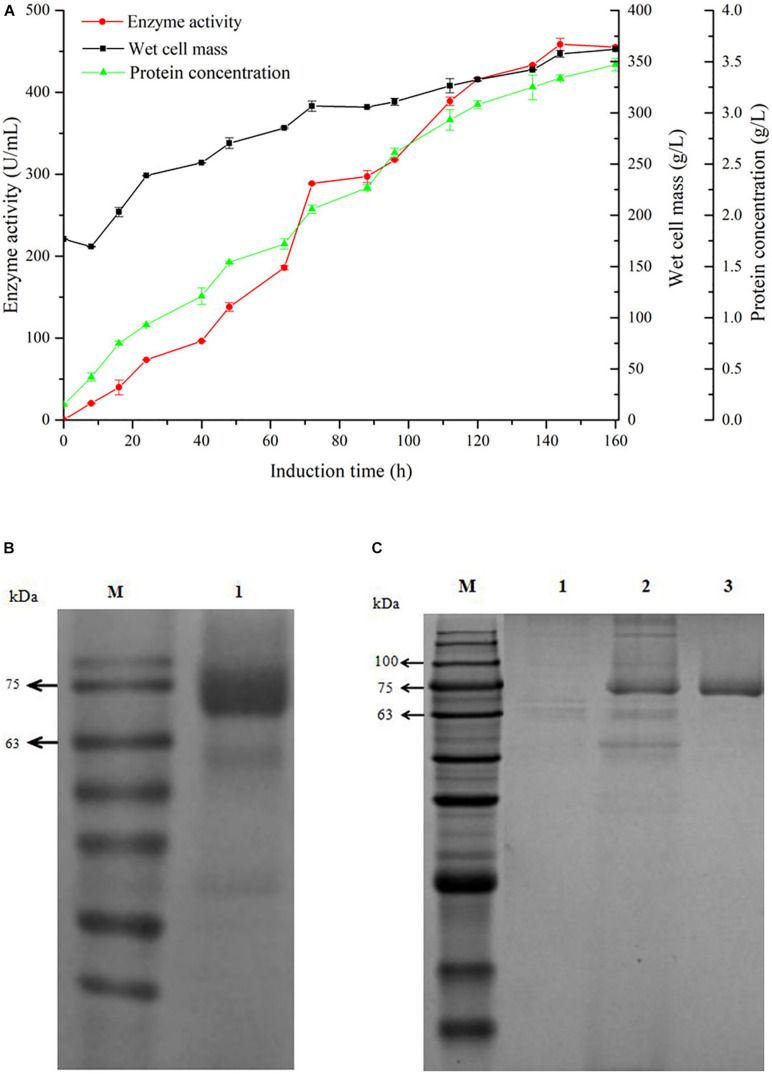
Time-course of GOxP_5_ in 20 L fermenter **(A)** and SDS-PAGE analysis **(B)** of recombinant glucose oxidase. The enzyme activity (•), wet cell mass (■), protein concentration (▲) were measured during high-cell-density fermentation. Line M, protein marker; line 1, purified GOxP_5_ fermentation supernatant withdrawn at 120 h after methanol induction. The supernatant samples after 96 h fermentation in shake flask of control strain pPICZα A/X33(line 1), the crude cell extract (line 2), and purified GOxP_5_ (line 3) were displayed by SDS-PAGE **(C)**.

For gaining high yield of GOxP_5_, the recombinant *P. pastoris* showing highest enzyme production capacity in shake flasks (35 U/mL) was further enlarged in a 20 L fermentor. As shown in Figure 1A, the enzyme yield underwent a continuous accumulation process in the former 145 h, and reached 458.6 U/mL (crude protein, 3.34 g/L) at this time point, which was about 12 times higher than the maximal enzymatic activity yield in shake flask.

### Purification of GOxP_5_ and proGOxP_5_

GOx was easily purified from *P. pastoris* culture supernatants using Ni-affinity chromatography owing to the correct exposure of the 6x His tag to the recombinant enzyme. After fivefold purification with a final yield of 50%, SDS-PAGE of the purified recombinant GOxP_5_ indicated that the Mw of a single band was around 70 kDa (Figures 1B,C).

### Properties of Recombinant GOxP_5_ and proGOxP_5_

**FIGURE 2:**
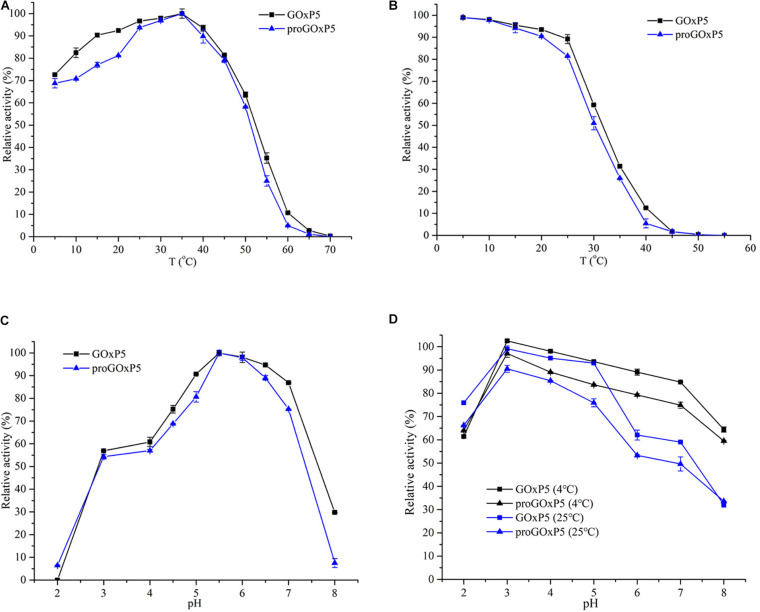
Determination of optimum temperature **(A)**, thermostability **(B)**, optimum pH **(C)**, and pH stability **(D)** of purified GOxP_5_ and proGOxP_5_. The optimal temperature was determined at temperatures ranging from 4 to 70°C in 50 mM sodium phosphate sodium citrate buffer (pH 6.0) **(A)**. For the analysis of thermostability, the purified enzymes were incubated in 50 mM sodium phosphate (pH6.0) for 5 min at 40–60°C **(B)**. The initial enzyme (without incubation) activity of each enzyme was defined as 100%. All the experiments were done in triplicates and the error bars indicate standard deviations. Optimal pH was measured in different buffers such as pH 2.0–3.0 (0.05 M glycine-HCl), pH 4.0–6.0 (0.05 M NaAc-HAc), and pH 7.0–8.0 (0.05 M Tris-HCl) **(C)**. For pH stability analysis, the enzymes were incubated with the above buffers for 120 min at 4 and 25°C **(D)**.

The optimal temperature and thermal stability of recombinant GOx are shown in Figures 2A,B. GOxP_5_ and proGOxP_5_ had the same optimal temperature at 35°C. GOxP_5_ could retain more than 80% of the highest activity at 10–45°C, and even at 4°C, it maintained 72.6% of the highest enzyme activity. In contrast, proGOxP_5_ retained 80% of its enzyme activity in only a narrow temperature range of 20–40°C. Recombinant GOxP_5_ and proGOxP_5_ exhibited the same optimum activity at pH 5.5 and could retain more than 50% activity over the pH range of 3.0–7.0 (Figures 2C,D). For pH stability, both enzymes retained more than 60% of the maximum activity from pH 2.0–5.0 after incubating at 25°C for 2 h, with GOxP_5_ remaining above 70%. In addition, it could retain more than 50% of the original activity after a 2 h incubation, even at pH 6.0–7.0. Recombinant GOxP_5_ displayed better acidic and neutral stability.

Several ions and chemical reagents had similar activation or inhibitory effects on the enzyme activity of purified GOxP_5_ and proGOxP_5_ as shown in Supplementary Figure 2. Most additives, such as K^+^, Na^+^, Mg^2+^, Zn^2+^, Fe^3+^, SDS, and EDTA, could activate the recombinant enzymes to enhance enzyme activity. Further, 5 mM Na^+^, Fe^3+^, and SDS caused 10–15% enhancement. Result showed that test ions and reagents had little inhibitory effect on these two enzymes with the exception of Fe^2+^, Ag^+^, and Cu^2+^. Purified recombinant GOxP_5_ and proGOxP_5_ displayed the maximum activity with D-glucose (100%) as a substrate and exhibited high substrate specificity (Supplementary Table 1). They exhibited no activity with substrates such as D-fructose, D-raffinose, D-arabinose, D-mannitol, lactose, and sucrose. They exhibited low reactivity (1–20%) to D-maltose, stachyose, D-galactose, D-mannose, D-sorbitol, and D-xylose. Therefore, glucose was used as the substrate to determine the kinetic parameters.

The K_m_ values of GOxP_5_ and proGOxP_5_ were 65.7 and 28 mM, respectively, which was similar to those of other previously reported fungal GOxs (Table 2). Though a higher K_m_ value contributed to a lower affinity for β-D-glucose from this GOx, it was also related to substrate concentration (Guo et al., 2010). The k_cat_/K_m_ of GOxP_5_ was 2086.758 mol^–1^⋅L⋅s^–1^, which was about twice that of proGOxP_5_, and it better reflected the excellent catalytic efficiency of GOxP_5_. Interestingly, as a whole, the activity and stability of GOxP_5_ was better than that of proGOxP_5_, which, according to Han et al. (2018) could be due to the signal peptide.

**TABLE 2:** Kinetic parameters of GOxP_5_ and proGOxP_5_.

Sample	Specific activity (U⋅mg^–1^)	K_m_ (mol⋅L^–1^)	V_max_ (μ mol⋅min^ –1^⋅mg^–1^)	k_cat_ (s^–1^)	k_cat_/K_m_ (mol^–1^⋅L⋅s^–1^)
proGOxP_5_	34.19	0.0280	29.114	31.714	1132.642
GOxP_5_	149.05	0.0657	125.866	137.1	2086.758

### Docking Analysis of GOxP_5_ and β-D-Glucose

**FIGURE 3:**
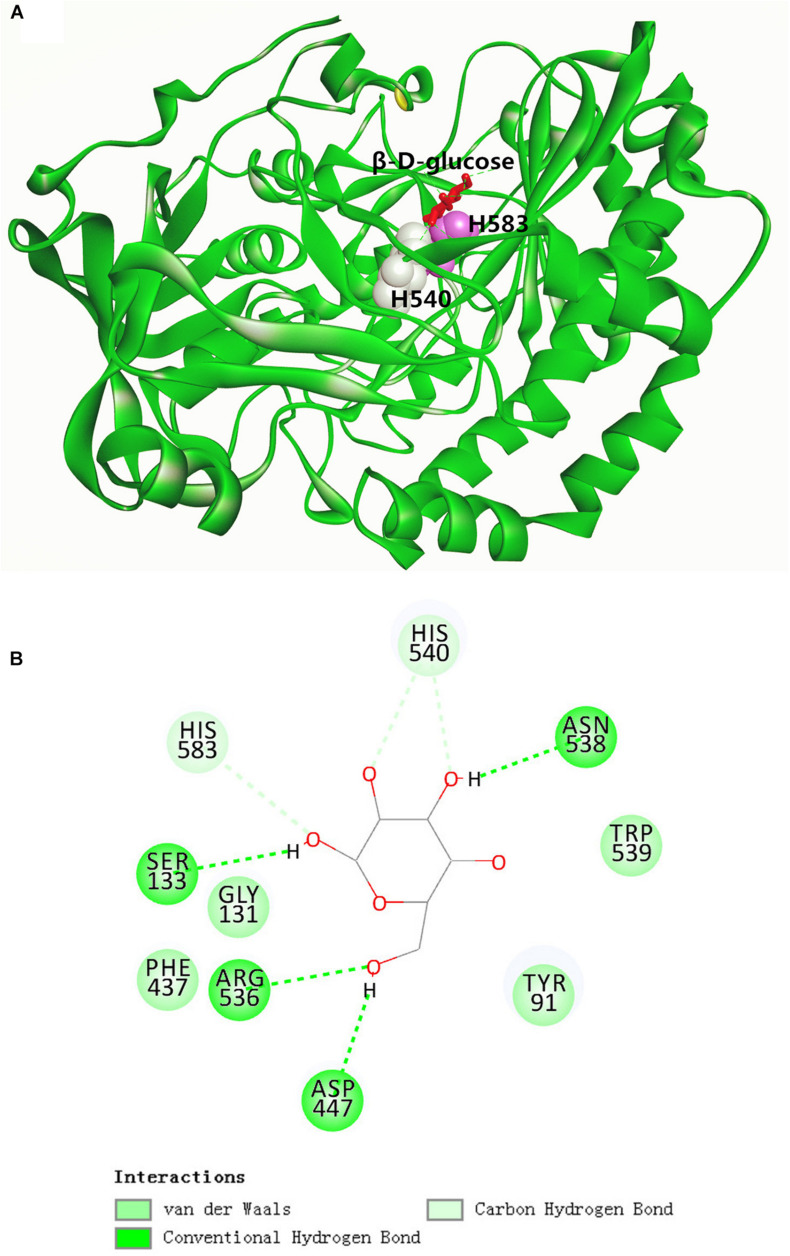
Overall structure of GOxP_5_ in complex with β-D-glucose **(A)**. Two-dimensional diagrams of non-covalent interactions of β-D-glucose/GOxP_5_ docking complex **(B)**.

As depicted in the docking complex of GOxP_5_ and β-D-glucose (Figure 3A), the glucose could properly dock into the catalytic cavity of the homologous GOxP_5_ model. Two catalytic amino acid sites, His_540_ and His_583_, located in two adjacent random coils, function as the proton donor and proton acceptor, respectively. The glucose could be fastened into the GOxP_5_ catalytic domain by forming non-covalent interactions with surrounding residues. As indicated in Figure 3B, β-D-glucose could form four conventional hydrogen bonds with Ser_133_, Arg_447_, Arg_536_, and Asn_538_, and two carbon hydrogen bonds with His_540_ and His_583_. Moreover, Van der Waals interactions surrounding the binding sites of β-D-glucose also greatly contribute to its stable binding in the correct position.

### Antibacterial Evaluation of GOxP_5_

**FIGURE 4:**
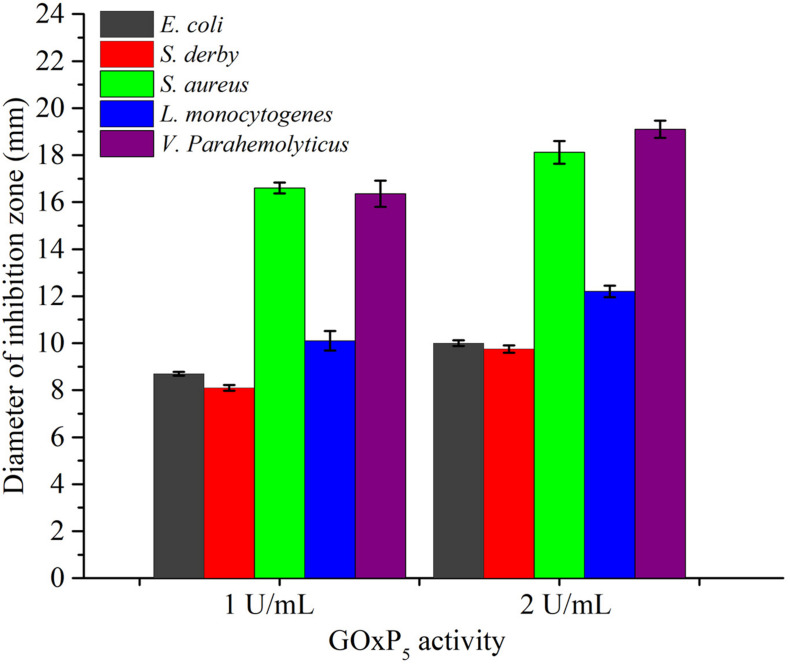
The average diameter of inhibition zones of GOxP_5_ against five bacterial strains (*E. coli*, *S. derby*, *S. aureus*, *V. parahemolyticus*, *L. monocytogenes*).

Using diffusion assays [zone of inhibition (ZOI) experiments], the recombinant GOxP_5_ (2 U/mL) induced 18.12, 12.2, and 19.1 mm ZOI diameters against of *S. aureus, L. monocytogenes*, and *V. parahaemolyticus*, respectively (Figure 4). While *V. parahaemolyticus* is Gram-negative strain, its growth was significantly inhibited as judged from MIC values (0.48 U/mL). At the same time, MIC value of GOxP_5_ were best against Gram-negative bacteria (*S. derby and E. coli*) at 0.96 U/mL. However, its seems that the 2 Gram-positive strains (*L. monocytogenes and S. aureus*) which were tested had a lower MIC than the 3 Gram-negative ones (Roy, 2009). Furthermore, the MIC value of GOxP_5_ for *S. aureus and L. monocytogenes* was 0.24 U/mL; of note, against *S. derby* and *E. coli* the MIC was up to 0.96 U/mL (Table 3). Interestingly, the determined MBC was found to be twice the MIC.

**TABLE 3:** MIC (U/mL) and MBC (U/mL) data of GOxP_5._

Bacteria	*E. coli*	*S. derby*	*S. aureus*	*L. monocytogenes*	*V. Parahemolyticus*
MIC	0.96	0.96	0.24	0.24	0.48
MBC	1.20	1.20	0.48	0.48	0.96

**FIGURE 5:**
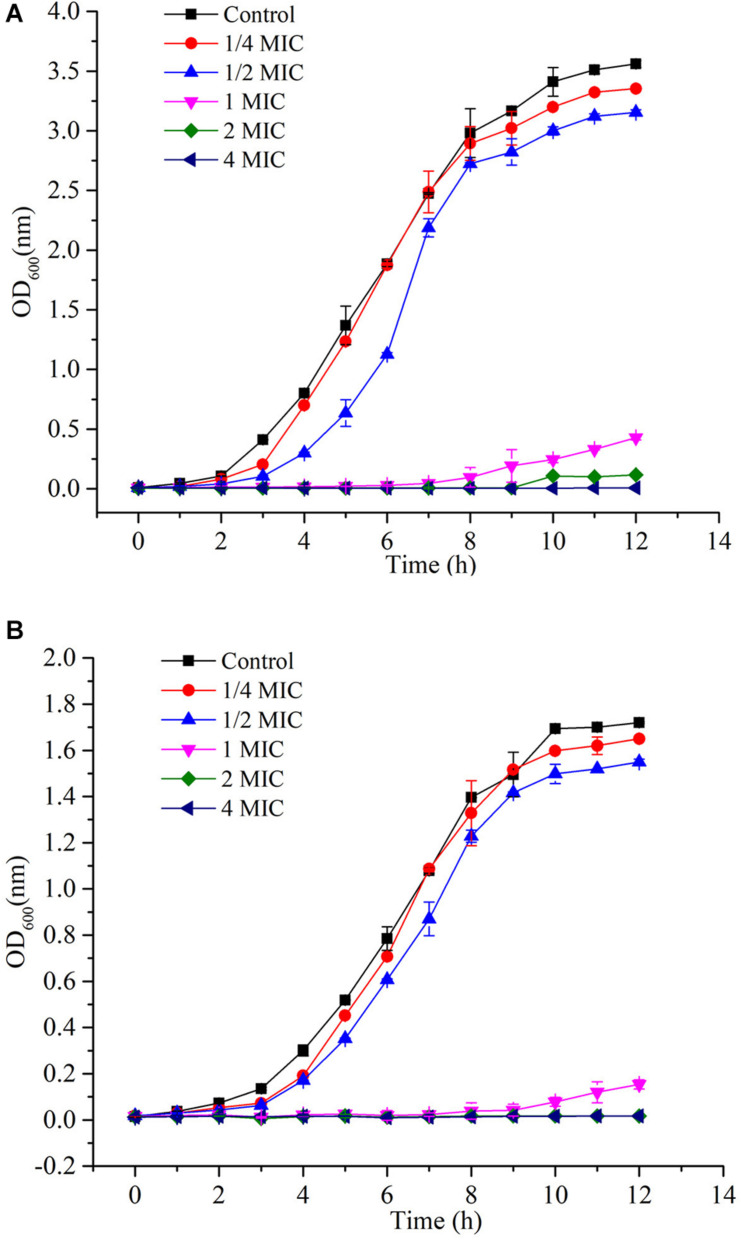
Growth curves **(A)** *L. monocytogenes* and **(B)** *V. parahemolyticus*.

**FIGURE 6:**
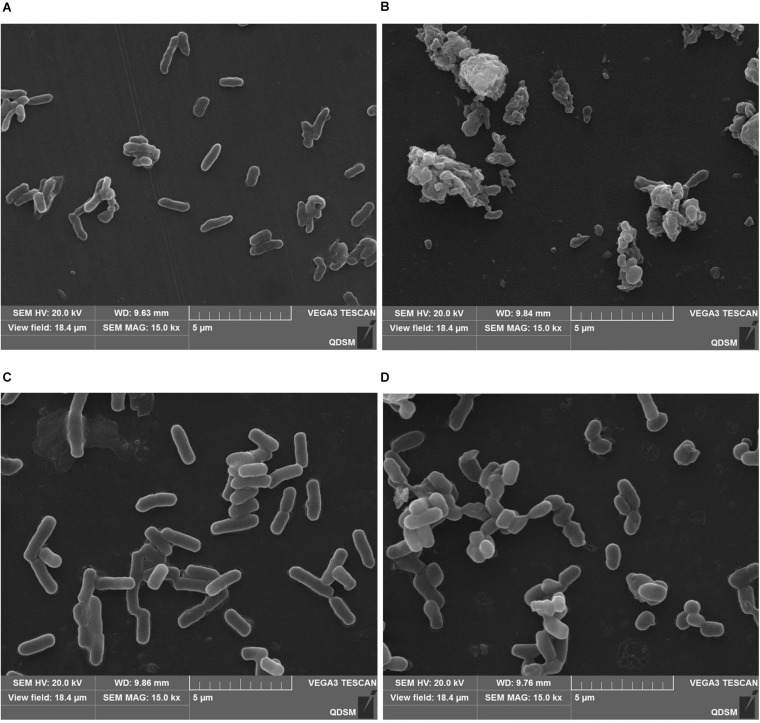
Scanning electron microscopic (SEM) images showing effect of GOxP_5_ on *L. monocytogenes* **(B)** and *V. parahemolyticus* **(D)**. **(A,C)** represents the blank control.

Since *L. monocytogenes* and *V. parahemolyticus* are common pathogenic bacteria in aquatic products, the effect of GOxP_5_ on their antibacterial growth curves was further determined. Importantly, the growth curves reflected the inhibitory effect of different GOxP_5_ concentrations on *L. monocytogenes* and *V. parahemolyticus* (Figures 5A,B), When a GOxP_5_ concentration lower than the MIC was used, the exponential phase was shorter than that in control bacterial cultures. On the other hand, when the concentration exceeded the MIC, the bacterial growth was completely inhibited. Furthermore, SEM images showed that GOxP_5_ (MIC concentration) caused different effects on *L. monocytogenes* and *V. parahemolyticus.* After 2 h of inoculation with GOxP_5_, the morphology of *L. monocytogenes* was destroyed, the cell membrane shrank and ruptured, and the cells stacked and adhered (Figure 6B). In comparison, the *V. parahemolyticus* cell membrane remained smooth, while the cells stacked and became shorter (Figure 6D).

## Effect of GOxP_5_ on Fish Preservation

### Changes in Sensory Quality

**FIGURE 7:**
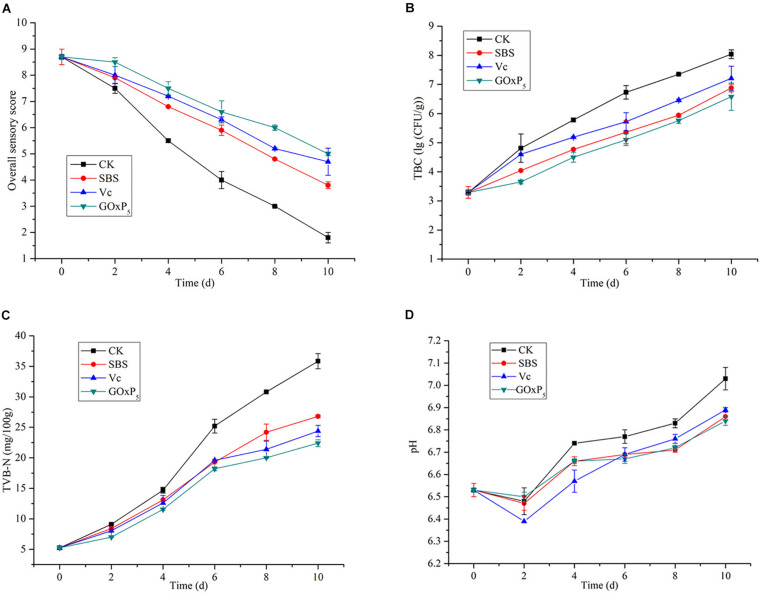
Changes in sensory quality **(A)**, total bacterial counts **(B)**, TVB-N **(C)**, and pH **(D)** values of grass carp filets with different treatments of preservatives during storage at 4°C. The vertical bars represent the standard deviations (*n* = 2).

In the grass carp preservation experiment, overall sensory quality scores for samples stored at 4°C are presented in Figure 7A, based on color, taste, odor, elasticity, and texture. Initially, the filets were fresh, their sensory scores exhibited a significant decline from an initial value of 9. The most obvious decline occurred in the CK group, which showed the SBS, Vc, and GOxP_5_ treatment groups were significantly better than the CK group. On the fourth day, the sensory score of the CK group was 5.5, which was lower than the acceptable value. However, the SBS and Vc scores reached an unacceptable level on the eighth (4.8) and tenth (4.7) days, respectively. Additionally, the GOxP_5_ treatment group scored 5 on the tenth day, which was still acceptable at the end of storage.

### Changes in Microbiological Quality

The initial TBC of all samples was near (3.29 ± 0.1) log CFU/g (Figure 7B). However, as the storage time of the fish filets in the four treatment groups increased, the TBC showed a distinctly different upward trend. On second day of storage, the TBC in the CK sample increased rapidly and reached (4.81 ± 0.09) log CFU/g. However, SS, Vc, and GOx only reached 4.04, 4.5, and 3.65 log CFU/g, respectively. After 4.46 days of storage, the TBC for the CK group reached the upper acceptable value for freshwater species by ICMSF (1986), whereas the GOxP_5_ samples were within the acceptable range until 8.59 days. SBS and GOxP_5_ samples had 6.88 and 6.58 log CFU/g, respectively, at 4°C for TBC on the tenth day of storage but CK reached 8.04 log CFU/g.

### Changes in Physicochemical Quality

TVB-N is mainly composed of volatile alkaline nitrogen-containing compounds such as ammonia and amines, which are formed as a result of the degradation of protein and non-protein nitrogenous compounds by microorganisms and enzymes. All groups showed increased TVB-N values as the storage time increased (Figure 7C); initially, the TVB-N of the fresh samples was 5.25 mg/100 g. On the sixth day, the TVB-N value in the control group reached 25.2 ± 1.13 mg/100 g, which exceeded the limit of the secondary freshness. Until 10 days, the GOxP_5_ treatment was lower than the commercially acceptable limit of 30 mg/100 g (Ocaño-Higuera et al., 2011), whereas the control reached nearly 35.85 ± 0.45 mg/100 g.

Changes in the sample pH values during storage are presented in Figure 7D. The same trend in the pH with storage time was observed in all grass carp filets. The pH of all samples decreased in the first 2 days of the storage period. Then, the pH of the CK, SBS, Vc, and GOxP_5_ groups increased from the initial 6.53–7.03, 6.86, 6.89, and 6.84, respectively, at the end of the 10 days storage period. GOxP_5_ was most effective in delaying the pH increase compared to the SBS and Vc groups.

## Discussion

As a biopreservative, GOx has great application value in food preservation. However, cold active was a key limitation for GOx in fish preservative applications, and few reports have used new GOx with excellent enzyme activity to extend aquatic product shelf life. In our study, a new cold-active GOx was isolated and successfully expressed in *P. pastoris*, and the biochemical and antimicrobial characteristics of recombinant enzymes were comprehensively analyzed, which proved it has significant effects on the preservation of grass carp.

Zeocine-based liquid PTVA method for selecting high copy number clones has been verified an efficient way for constructing high growth rate and high protein yield stains (Aw and Polizzi, 2016). This result was in accordance with previous report that high-cell-density fermentation of *P. pastoris* normally enlarged the enzyme yield by ten folds (Li et al., 2007; Figure 1A). After that, the total enzyme yield and wet cell mass tended to decrease, and the methanol consumption rate apparently reduced, which implied the deadline of fermentation. The maximal enzymatic activity of GOxP_5_ in shake flask was much higher than many heterologously expressed enzymes in *P. pastoris*, such as GOx form *P. variable* (0.33 U/mL), *A. niger* (1.23 U/mL), and *A. niger* NRLL-3 (17.5 U/mL) (Kalisz et al., 1997; Yamaguchi et al., 2007; Kovačević et al., 2014). Although the highest expression level of GOxP_5_ in *P. pastoris* after high-cell-density fermentation was lower than that of GOD-m from *Penicillium notatum* (615 U/mL) (Gao et al., 2012), it was still of great value due to its excellent characteristics in food preservation area.

The optimum reported temperature range for most GOxs was from 25 to 60°C (Table 4). GOxP_5_, with an optimum temperature of 35°C, in particular maintained 72.6% of the highest enzyme activity even at 4°C. However, most reported fungal GOxs retained less than 70% of its maximum activity at 4°C, which demonstrated GOxP_5_ had a higher activity at refrigeration temperature. Tu et al. (2019) showed that the activity of *Aspergillus* GOx at 20°C was approximately 75% of the highest activity. Although Sukhacheva et al. (2004) discovered that GOx from *Penicillium* could maintain about 80% at 20°C, enzyme activity reduced rapidly with decreasing temperature. When the temperature dropped to 10–15°C, its activity decreased significantly to 40% (Witt et al., 1998; Courjean and Mano, 2011), which showed it is difficult to exert effective enzyme activity at 4°C. However, a preservative that could effectively function at 4°C was required in the preservation of aquatic products especially fish. In addition, the pH value of most perishable foods such as meat, fish and shrimps was close to neutral, and GOxP_5_ could play a good role in this pH value range (Figure 2C). When treated at pH = 7 and 4°C for 2 h, the enzyme activity only lost 20%, which further showed that it could work effectively under low temperature conditions (Figure 2D).

**TABLE 4:** Comparison of the properties of GOxP_5_ with different fungal GOxs.

GOx source	Temperature optimum (°C)	Cold adapted	pH optimum	K_m_ (mM)	k_cat_ /K_m_ (mM^–1^⋅s^–1^)	References
*Penicillium* sp.	30	>72% activity at 5°C	5.5	65.7	2.09	This study
*Aspergillus niger* (mutant M4)	40	About 75% activity at 20°C	6.0	32.82	32.82	Tu et al., 2019
*Aspergillus tubingensis*	60	<40% activity at 30°C	4.5	53.23	11.32	Kriaa et al., 2015
*Penicillium amagasakiense*	28	33.3% activity at 15°C	7.0	–	–	Witt et al., 1998
*Penicillium amagasakiense*	40–50	<40% activity at 10°C	4.5–6.5	6.2	323	Kalisz et al., 1997; Courjean and Mano, 2011
*Penicillium funiculosum* 433	30	About 80% activity at 20°C	6.0–8.6	3.3	–	Sukhacheva et al., 2004
*Penicillium variabile* P16	45	About 60% activity at 30°C	6.0	15.25	5.58 × 10^4^	Crognale et al., 2006

Their strong resistance to most metal ions and chemical reagents makes wide application potential for the food industry. The molecular weight of purified recombinant GOxP_5_ was approximately 6.14 kDa larger than the theoretical Mw (Figure 1B). This demonstrated that N-glycosylation sites on the protein surface were glycosylated during post-translational processing in *P. pastoris* (Kalisz et al., 1997). Research reports have shown that glycosylation may have an effect on the catalytic ability, but the influence was not obvious in this experiment (Crognale et al., 2006; Kohen et al., 1997). In addition, a comparative study indicated that the wild-type signal peptide apparently influenced the GOx expression level and biochemical properties. Specifically, recombinant proGOxP_5_ (containing native signal peptide) decreased the enzyme activity by nearly five times and only retained 62% of the maximum activity at 4°C (Figure 2). Consistent with the research of Chang et al. (2011), the N-terminal signal peptide had a negative effect on gene expression.

Since GOx can lead to the production of gluconic acid and H_2_O_2_, reducing the pH, it indirectly possesses significant antimicrobial activity against different foodborne pathogens (Pluschkell et al., 1996; Bankar et al., 2009). Consistently with reported studies, GOx had a good antimicrobial effect on the common spoilage bacteria in food, and the antibacterial effect on *L. monocytogenes* and *V. parahemolyticus* was in different degree. As Gram-negative bacteria, *V. parahaemolyticus* was generally more resistant to antimicrobial biomolecules due to its lipopolysaccharide outer membrane (Roy, 2009). The result of SEM images confirmed that GOxP_5_ destroys the cell membrane integrity of specific bacteria (Figure 6), which further indicated its good antibacterial effect (Bankar et al., 2009).

In the experiment of fish preservation, sensory, microbiological analyses, and physicochemical changes of grass carp filets were evaluated during 10 days of storage at 4°C (Figure 7). Results showed GOxP_5_ had an excellent freshness preservation effect compared to the SBS and Vc. In particular, GOxP_5_ had a better effect on sensory quality. The ammonia, fishy, and putrid odors were light in the GOxP_5_ group, and these unpleasant odors were due to the spoilage of microorganisms that cause the accumulation of trimethylamine and biological amines (Field et al., 1986). The TBC of the CK group was significantly higher than the treated groups (SBS, Vc, and GOxP_5_). Although SBS had been considered as antimicrobial food additive and it was concluded that it decreases total bacteria (Omar, 1998), it was still not as advantageous as GOxP_5._ The significant difference between GOxP_5_ and the other three treatments can be owing to the strong inhibitory effect of released H_2_O_2_. At the same conditions, the GOxP_5_ treatment group could be extended for 3 days based on the TVB-N analyses. The results showed that pH values in all fish samples decreased initially before increasing. Researchers indicated that the initial pH decreases could be associated with lactic acid during anaerobic glycolysis and inorganic phosphate released by ATP degradation, and the subsequent pH increase might be attributed to amine and trimethylamine accumulation (Liu et al., 2013; Yu et al., 2017). Therefore, the shelf life of grass carp could be significantly extended after GOxP_5_ treatment.

Since GOxP_5_ had an excellent effect compared to traditional chemical preservatives mentioned before, it is promising for its application for the biopreservation of high added-value perishable foods such as fresh fish filets or seafood. Previous studies demonstrated that the application of composite biological preservatives (lysozyme, nisin, and others) in aquatic products could more effectively extend the shelf life than a single preservative. Morsy et al. (2018) provided a combination of nisin, lysozyme, and ZnO nanoparticles effectively inhibiting *Escherichia coli*, *Bacillus cereus*, and *Listeria monocytogenes* and increasing refrigerated beef safety. In addition, Ge et al. (2012) immobilized GOx in an electrospun nanofibrous membrane, which had obvious bacteriostatic activity against bacteria for food preservation. Thus, the cold-active GOxP_5_ has great application value in grass carp preservation as a biopreservative in this study, the application scope could be further extended by combing with other preservatives.

## Data Availability Statement

The datasets presented in this study can be found in online repositories. The names of the repository/repositories and accession number(s) can be found in the article/Supplementary Material.

## Author Contributions

MY: investigation, data curation, writing-original draft preparation, and methodology. CN: methodology and writing-review. SY: visualization and formal analysis. QL: software and data curation. ZL: conceptualization and data curation. HM: writing-review, editing, supervision, funding acquisition, and project administration. All authors contributed to the article and approved the submitted version.

## Conflict of Interest

The authors declare that the research was conducted in the absence of any commercial or financial relationships that could be construed as a potential conflict of interest.

## Footnotes

1 http://www.ncbi.nlm.nih.gov/BLAST

2 http://www.sbg.bio.ic.ac.uk/phyre2/html/page.cgi?id=index

## Abbreviations

GOx: glucose oxidase
FAD: flavin adenine dinucleotide
CTAB: cetyl trimethyl ammonium bromide
SDS-PAGE: Sodium dodecyl sulfate-polyacrylamide gel electrophoresis
ZOI: zone of inhibition
TBC: total bacterial count
TVB-N: total volatile basic nitrogen
MIC: minimum inhibitory concentration
MBC: the minimum bactericidal concentration.

## References

[B1] Asad M. Arshad M. N. Oves M. Khalid M. Khan S. A. Asiri A. M. (2020). N-Trifluoroacetylated pyrazolines: Synthesis, characterization and antimicrobial studies. *Bioorg. Chem.* 99:103842. 10.1016/j.bioorg.2020.103842 32315898

[B2] Aw R. Polizzi K. M. (2016). Liquid PTVA: a faster and cheaper alternative for generating multi-copy clones in *Pichia pastoris* Microb. *Cell Fact.* 15 1–11. 10.1385/0-89603-421-6:1 PMC4744420 26849882

[B3] Bankar S. B. Bule M. V. Singhal R. S. Ananthanarayan L. (2009). Glucose oxidase – an overview. *Biotechnol. Adv.* 27 489–501. 10.1016/j.biotechadv.2009.04.003 19374943

[B4] Başyiǧit B. Saǧlam H. Kandemir Ş Karaaslan A. Karaaslan M. (2020). Microencapsulation of sour cherry oil by spray drying: evaluation of physical morphology, thermal properties, storage stability, and antimicrobial activity. *Powder Technology.* 364 654–663. 10.1016/j.powtec.2020.02.035

[B5] Bradford M. M. (1976). A rapid and sensitive method for the quantitation of microgram quantities of protein utilizing the principle of protein-dye binding. *Anal. Biochem.* 72 248–254. 10.1016/0003-2697(76)90527-3 942051

[B6] Chang S. W. Li C. F. Lee G. C. Yeh T. Shaw J. F. (2011). Engineering the expression and biochemical characteristics of recombinant candida rugosa LIP2 lipase by removing the additional n-terminal peptide and regional codon optimization. *J. Agric. Food Chem.* 59 6710–6719. 10.1021/jf200537w 21561168

[B7] Courjean O. Mano N. (2011). Recombinant glucose oxidase from *Penicillium amagasakiense* for efficient bioelectrochemical applications in physiological conditions. *J. Biotechnol.* 151 122–129. 10.1016/j.jbiotec.2010.10.077 21040747

[B8] Crognale S. Pulci V. Brozzoli V. Petruccioli M. Federici F. (2006). Expression of Penicillium variabile P16 glucose oxidase gene in Pichia pastoris and characterization of the recombinant enzyme. *Enzyme Microb. Technol.* 39 1230–1235. 10.1016/j.enzmictec.2006.03.005

[B9] Dondero M. Egaña W. Tarky W. Cifuentes A. Torres J. A. (1993). Glucose oxidase/Cqtalase improves preservation of shrimp (Heterocarpus reedi). *J. Food Sci.* 58 774–779. 10.1111/j.1365-2621.1993.tb09356.x

[B10] European Food Safety Authority (2009). General approach to fish welfare and to the concept of sentience in fish – scientific opinion of the panel on animal health and welfare. Adopted on 29 January 2009. *EFSA J.* 954 1–27.

[B11] Field C. E. Pivarnik L. F. Barnett S. M. Rand A. G. (1986). Utilization of glucose oxidase for extending the shelf−life of fish. *J. Food Sci.* 51 66–70. 10.1111/j.1365-2621.1986.tb10837.x

[B12] Gao Z. Li Z. Zhang Y. Huang H. Li M. Zhou L. (2012). High-level expression of the *Penicillium notatum* glucose oxidase gene in *Pichia pastoris* using codon optimization. *Biotechnol. Lett.* 34 507–514. 10.1007/s10529-011-0790-6 22052258

[B13] Ge L. Zhao Y. S. Mo T. Li J. R. Li P. (2012). Immobilization of glucose oxidase in electrospun nanofibrous membranes for food preservation. *Food Control* 26 188–193. 10.1016/j.foodcont.2012.01.022

[B14] Guo Y. Lu F. Zhao H. Tang Y. Lu Z. (2010). Cloning and heterologous expression of glucose oxidase gene from Aspergillus niger Z-25 in Pichia pastoris. *Appl. Biochem. Biotechnol.* 162 498–509. 10.1007/s12010-009-8778-6 19784554

[B15] Han B. Hou Y. Jiang T. Lv B. Zhao L. Feng X. (2018). Computation-aided rational deletion of C-terminal region improved the stability, activity, and expression level of GH2 β-glucuronidase. *J. Agric. Food Chem.* 66 11980–11389. 10.1021/acs.jafc.8b03449 30296070

[B16] Hatzinikolaou D. G. Hansen O. C. Macris B. J. Tingey A. Kekos D. Goodenough P. (1996). A new glucose oxidase from *Aspergillus niger*: Characterization and regulation studies of enzyme and gene. *Appl. Microbiol. Biotechnol.* 46 371–381. 10.1007/s002530050832 8987726

[B17] ICMSF (1986). “(International commission on microbiological specifications for foods). Sampling plans for fish and shellfish,” in *Sampling for Microbiological Analysis: Principles and Scientific Applications: ICMSF, Microorganisms in Foods*, 2 Edn, Vol. 2 (Toronto, ON: University of Toronto Press), 181–196.

[B18] Jeon Y. J. Kamil J. Y. V. A. Shahidi F. (2002). Chitosan as an edible invisible film for quality preservation of herring and Atlantic cod. *J. Agric. Food Chem.* 50 5167–5178. 10.1021/jf011693l 12188625

[B19] Kalisz H. M. Hendle J. Schmid R. D. (1997). Structural and biochemical properties of glycosylated and deglycosylated glucose oxidase from *Penicillium amagasakiense*. *Appl. Microbiol. Biotechnol.* 47 502–507. 10.1007/s002530050963 9210339

[B20] Kelley L. A. Mezulis S. Yates C. M. Wass M. N. Sternberg M. J. E. (2015). The phyre2 web portal for protein modeling, prediction and analysis. *Nat. Protoc.* 10 845–858. 10.1038/nprot.2015.053 25950237 PMC5298202

[B21] Kohen A. Jonsson T. Klinman J. P. (1997). Effects of protein glycosylation on catalysis: Changes in hydrogen tunneling and enthalpy of activation in the glucose oxidase reaction. *Biochemistry* 36 2603–2611. 10.1021/bi962492r 9054567

[B22] Kovačević G. Blažić M. Draganić B. Ostafe R. Gavrović-Jankulović M. Fischer R. (2014). Cloning, heterologous expression, purification and characterization of M12 mutant of Aspergillus niger glucose oxidase in yeast Pichia pastoris KM71H. *Mol. Biotechnol.* 56 305–311. 10.1007/s12033-013-9709-x 24122283

[B23] Kriaa M. Hammami I. Sahnoun M. Azebou M. C. H. Triki M. A. l Kammoun R. (2015). Purification, biochemical characterization and antifungal activity of a novel Aspergillus tubingensis glucose oxidase steady on broad range of pH and temperatures. *Bioprocess Biosyst. Eng.* 38 2155–2166. 10.1007/s00449-015-1455-y 26280215

[B24] Kusai K. Sekuzu I. Hagihara B. Okunuki K. Yamauchi S. Nakai M. (1960). Crystallization of glucose oxidase from *Penicillium amagasakiense*. *BBA - Biochim. Biophys. Acta* 40 555–557. 10.1016/0006-3002(60)91406-2 14412928

[B25] Li P. Anumanthan A. Gao X. G. Ilangovan K. Suzara V. V. Düzgüneş N. (2007). Expression of recombinant proteins in *Pichia pastoris*. *Appl. Biochem. Biotechnol.* 142 105–124. 10.1007/s12010-007-0003-x 18025573

[B26] Liu D. Liang L. Xia W. Regenstein J. M. Zhou P. (2013). Biochemical and physical changes of grass carp (Ctenopharyngodon idella) fillets stored at -3 and 0 °C. *Food Chem.* 140 105–114. 10.1016/j.foodchem.2013.02.034 23578621

[B27] Liu M. Gong Y. Sun H. Zhang J. Zhang L. Sun J. (2020c). Characterization of a novel chitinase from sweet potato and its fungicidal effect against *Ceratocystis fimbriata*. *J. Agric. Food Chem.* 68 7591–7600. 10.1021/acs.jafc.0c01813 32585101

[B28] Liu Z. Ning C. Yuan M. Yang S. Wei X. Xiao M. (2020a). High-level expression of a thermophilic and acidophilic β-mannanase from Aspergillus kawachii IFO 4308 with significant potential in mannooligosaccharide preparation. *Bioresour. Technol.* 295:122257. 10.1016/j.biortech.2019.122257 31648129

[B29] Liu Z. Yuan M. Zhang X. Liang Q. Yang M. Mou H. (2020b). A thermostable glucose oxidase from *Aspergillus heteromophus* CBS 117.55 with broad pH stability and digestive enzyme resistance. *Protein Expr. Purif.* 176 105717. 10.1016/j.pep.2020.105717 32745582

[B30] López-Luna J. Vásquez L. Torrent F. Villarroel M. (2013). Short-term fasting and welfare prior to slaughter in rainbow trout, *Oncorhynchus mykiss*. *Aquaculture* 400–401 142–147. 10.1016/j.aquaculture.2013.03.009

[B31] Macauley-Patrick S. Fazenda M. L. McNeil B. Harvey L. M. (2005). Heterologous protein production using the *Pichia pastoris* expression system. *Yeast* 22 249–270. 10.1002/yea.1208 15704221

[B32] Massa S. Petruccioli M. Brocchi G. F. Altieri C. Sinigaglia M. Spano G. (2001). Growth inhibition by glucose oxidase system of enterotoxic *Eseherichia coli* and *Salmonella* derby: in vitro studies. *World J. Microbiol. Biotechnol.* 17 287–291. 10.1023/A:1016690214114

[B33] Morsy M. K. Elsabagh R. Trinetta V. (2018). Evaluation of novel synergistic antimicrobial activity of nisin, lysozyme, EDTA nanoparticles, and/or ZnO nanoparticles to control foodborne pathogens on minced beef. *Food Control* 92 249–254. 10.1016/j.foodcont.2018.04.061

[B34] Ocaño-Higuera V. M. Maeda-Martínez A. N. Marquez-Ríos E. Canizales-Rodríguez D. F. Castillo-Yáñez F. J. Ruíz-Bustos E. (2011). Freshness assessment of ray fish stored in ice by biochemical, chemical and physical methods. *Food Chem.* 125 49–54. 10.1016/j.foodchem.2010.08.034

[B35] Ojagh S. M. Rezaei M. Razavi S. H. Hosseini S. M. H. (2010). Effect of chitosan coatings enriched with cinnamon oil on the quality of refrigerated rainbow trout. *Food Chem.* 120 193–198. 10.1016/j.foodchem.2009.10.006

[B36] Omar M. I. V. (1998). *Utilization of Sodium Metabisulphite for Preservation of Frozen-Thawed Shrimp (Pandaleus Borealis).* Reykjavik: United Nations University Fisheries Training Programme, 1–16.

[B37] Pluschkell S. Hellmuth K. Rinas U. (1996). Kinetics of glucose oxidase excretion by recombinant *Aspergillus niger*. *Biotechnol. Bioeng.* 51 215–220. 10.1002/(sici)1097-0290(19960720)51:2<215::aid-bit11>3.0.co;2-l 18624331

[B38] Roy H. (2009). Tuning the properties of the bacterial membrane with aminoacylated phosphatidyl glycerol. *IUBMB Life* 61 940–953. 10.1002/iub.240 19787708 PMC2757517

[B39] Sukhacheva M. V. Davydova M. E. Netrusov A. I. (2004). Production of *Penicillium funiculosum* 433 glucose oxidase and its properties. *Appl. Biochem. Microbiol.* 40 25–29. 10.1023/B:ABIM.0000010346.47923.6c 15029694

[B40] Sun X. Hong H. Jia S. Liu Y. Luo Y. (2020). Effects of phytic acid and lysozyme on microbial composition and quality of grass carp (*Ctenopharyngodon idellus*) fillets stored at 4°C. *Food Microbiol.* 86:103313. 10.1016/j.fm.2019.103313 31703873

[B41] Tang H. Yao B. Gao X. Yang P. Wang Z. Zhang G. (2016). Effects of glucose oxidase on the growth performance, serum parameters and faecal microflora of piglets. *S. Afr. J. Anim. Sci.* 46 14–20. 10.4314/sajas.v46i1.2

[B42] Trott O. Olson A. J. (2010). Autodock vina: improving the speed and accuracy of docking with a new scoring function, efficient optimization, and multithreading. *J. Comput. Chem.* 31 455–461. 19499576 10.1002/jcc.21334 PMC3041641

[B43] Tu T. Wang Y. Huang H. Wang Y. Jiang X. Wang Z. (2019). Improving the thermostability and catalytic efficiency of glucose oxidase from *Aspergillus niger* by molecular evolution. *Food Chem.* 281 163–170. 10.1016/j.foodchem.2018.12.099 30658743

[B44] Uchima C. A. Tokuda G. Watanabe H. Kitamoto K. Arioka M. (2012). Heterologous expression in pichia pastoris and characterization of an endogenous thermostable and high-glucose-tolerant β-glucosidase from the termite *Nasutitermes takasagoensis*. *Appl. Environ. Microbiol.* 78 4288–4293. 10.1128/AEM.07718-11 22522682 PMC3370526

[B45] Van Burik J. A. H. Schreckhise R. W. White T. C. Bowden R. A. Myerson D. (1998). Comparison of six extraction techniques for isolation of DNA from filamentous fungi. *Med. Mycol.* 36 299–303. 10.1046/j.1365-280X.1998.00161.x 10075499

[B46] Wang L. Liu F. Jiang Y. Chai Z. Li P. Cheng Y. (2011). Synergistic antimicrobial activities of natural essential oils with chitosan films. *J. Agric. Food Chem.* 59 12411–12419. 10.1021/jf203165k 22034912

[B47] Witt S. Singh M. Kalisz H. M. (1998). Structural and kinetic properties of nonglycosylated recombinant Penicillium amagasakiense glucose oxidase expressed in *Escherichia coli*. *Appl. Environ. Microbiol.* 64 1405–1411. 10.1128/aem.64.4.1405-1411.1998 9546178 PMC106162

[B48] Xu D. Sun L. Li C. Wang Y. Ye R. (2018). Inhibitory effect of glucose oxidase from *Bacillus* sp. CAMT22370 on the quality deterioration of Pacific white shrimp during cold storage. *LWT Food Sci. Technol.* 92 339–346. 10.1016/j.lwt.2018.02.025

[B49] Yamaguchi M. Tahara Y. Nakano A. Taniyama T. (2007). Secretory and continuous expression of Aspergillus niger glucose oxidase gene in *Pichia pastoris*. *Protein Expr. Purif.* 55 273–278. 10.1016/j.pep.2007.05.006 17590349

[B50] Yoo W. Rand A. G. (1995). Antibacterial effect of glucose oxidase on growth of *Pseudomonas* fragi as related to pH. *J. Food Sci.* 60 868–871. 10.1111/j.1365-2621.1995.tb06249.x

[B51] Yu D. Li P. Xu Y. Jiang Q. Xia W. (2017). Physicochemical, microbiological, and sensory attributes of chitosan-coated grass carp (*Ctenopharyngodon idellus*) fillets stored at 4°C. *Int. J. Food Prop.* 20 390–401. 10.1080/10942912.2016.1163267

