# Supplementary material for: A New Cold-Active Glucose Oxidase From Penicillium: High-Level Expression and Application in Fish Preservation
Source: Front Microbiol. 2020 Nov 23;11:606007. doi: 10.3389/fmicb.2020.606007 (PMC7719636; doi:10.3389/fmicb.2020.606007)
Supplement: Supplementary file 1 [file Data_Sheet_1.doc]

**Supplementary Table 1**

Substrate specificity of FAD-GOx activity. Dehydrogenase activity to various saccharides (1000 mM) was examined at pH 6.0 and 30°C. Activity to D-glucose was set as 100 %.

| Substrate | Relative activity (%)^a^ | |
| --- | --- | --- |
|  | proGOxP_5_ | GOxP_5_ |
| D-Glucose | 100 | 100 |
| D-Galactose | 5.34±1.13 | 10.32±0.89 |
| D-Mannose | 6.11±0.61 | 7.67±0.57 |
| D-Sorbitol | 1.15±0.53 | 3.54±0.25 |
| D-Xylose | 5.34±1.43 | 7.37±0.71 |
| D-Maltose | 17.56±0.91 | 21.83±1.23 |
| D-Fructose | ND | ND |
| D-Raffinose | ND | ND |
| D-Arabinose | ND | ND |
| D-Mannitol | ND | ND |
| Lactose | ND | ND |
| Sucrose | ND | ND |
| Stachyose | 14.12±2.31 | 13.57±1.59 |

^a^ Values represent the means ±SD (n =3) relative to the untreated control samples.

**Supplementary Table 2** Sensory evaluation standard of grass carp fillets

| Quality parameter | Score | | | | |
| --- | --- | --- | --- | --- | --- |
|  | 8-10 | 6-8 | 4-6 | 2-4 | 0-2 |
| Muscle tissue | Complete, clear texture, no juice exudative, solid and elastic | Slight clear texture, no juice exudative, elastic | Not tight, a small amount of juice exudative, slightly elastic | Loose, more juice exudative, slightly elastic | No clear texture, a lot of juice exudative, no elasticity |
| Surface color | Glossy appearance, bright surface | Slight glossy appearance, bright surface | Slight glossy appearance, dull surface | No Glossy appearance, yellow surface | No Glossy appearance, dim surface |
| Odor | No fishiness, no earthy smell | Little fishiness, no off-odor | Little freshness and off-odor | Distinct freshness and off-odors | Strong ammonia odors |


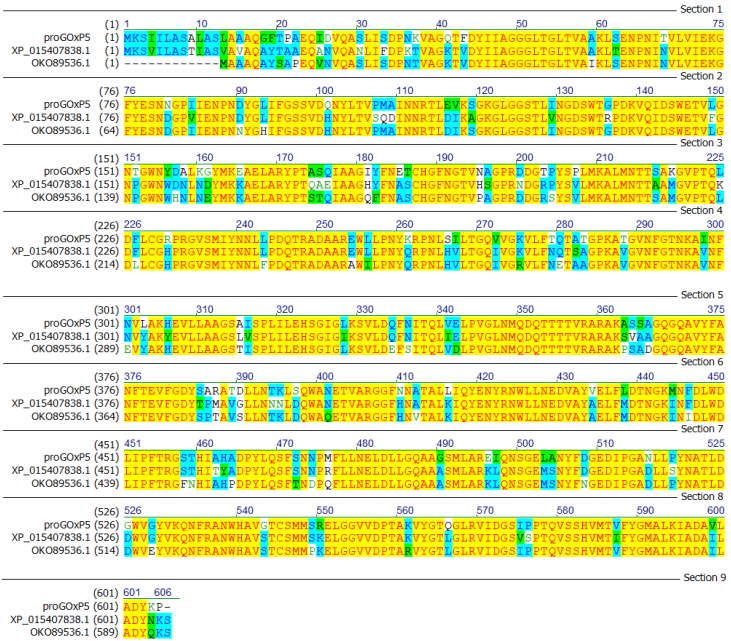


**Supplementary Figure 1.** Multiple analysis of amino acid sequences of GOxP_5_. Sequences listed include GOx from *Penicillium* in this study (MN912809), and two published glucose oxidases, *Aspergillus nomius* (XP_015407838.1) and *Penicillium subrubescens* (OKO89536.1). The two black triangles and stars indicated catalytic residues and FAD binding sites, respectively. The yellow columns indicated completely identical amino acid regions.


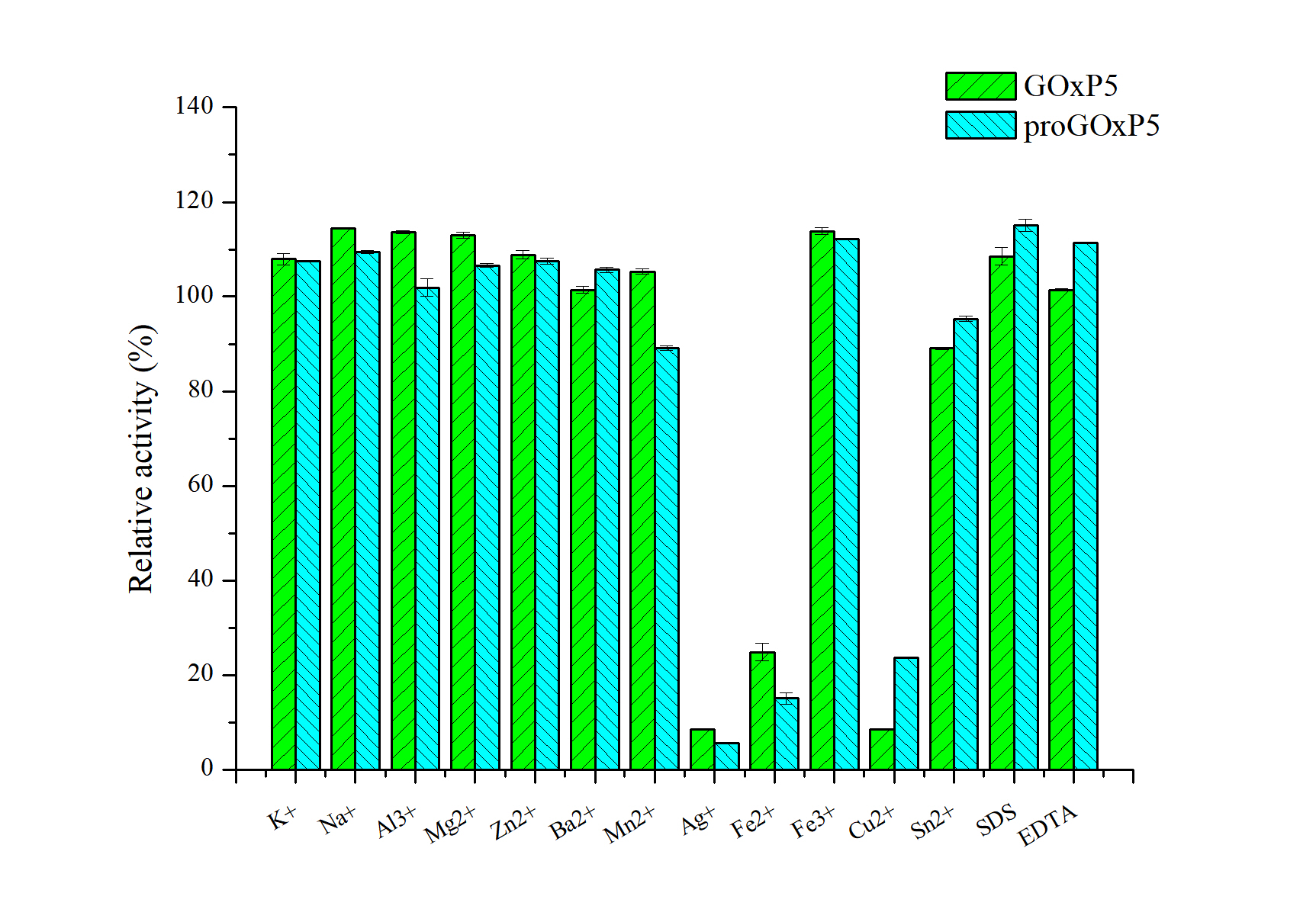


**Supplementary Figure 2.** Effects of ions and chemical reagents on the enzyme activity of GOxP_5_ and proGOxP_5_.
